# Supplementary material for: RNASeek: A Cross-Phyla Generative Foundation Model for Multipurpose RNA Modeling and Reinforcement Learning-Based Design
Source: bioRxiv. 2026 Sep 28:2026.09.24.754173. Preprint. [Version 1] doi: 10.64898/2026.09.24.754173 (PMC13648417; doi:10.64898/2026.09.24.754173)
Supplement: Supplementary file 1 [file NIHPP2026.09.24.754173v1-supplement-1.pdf]

Supplementary Figures

Supplementary Fig. 1

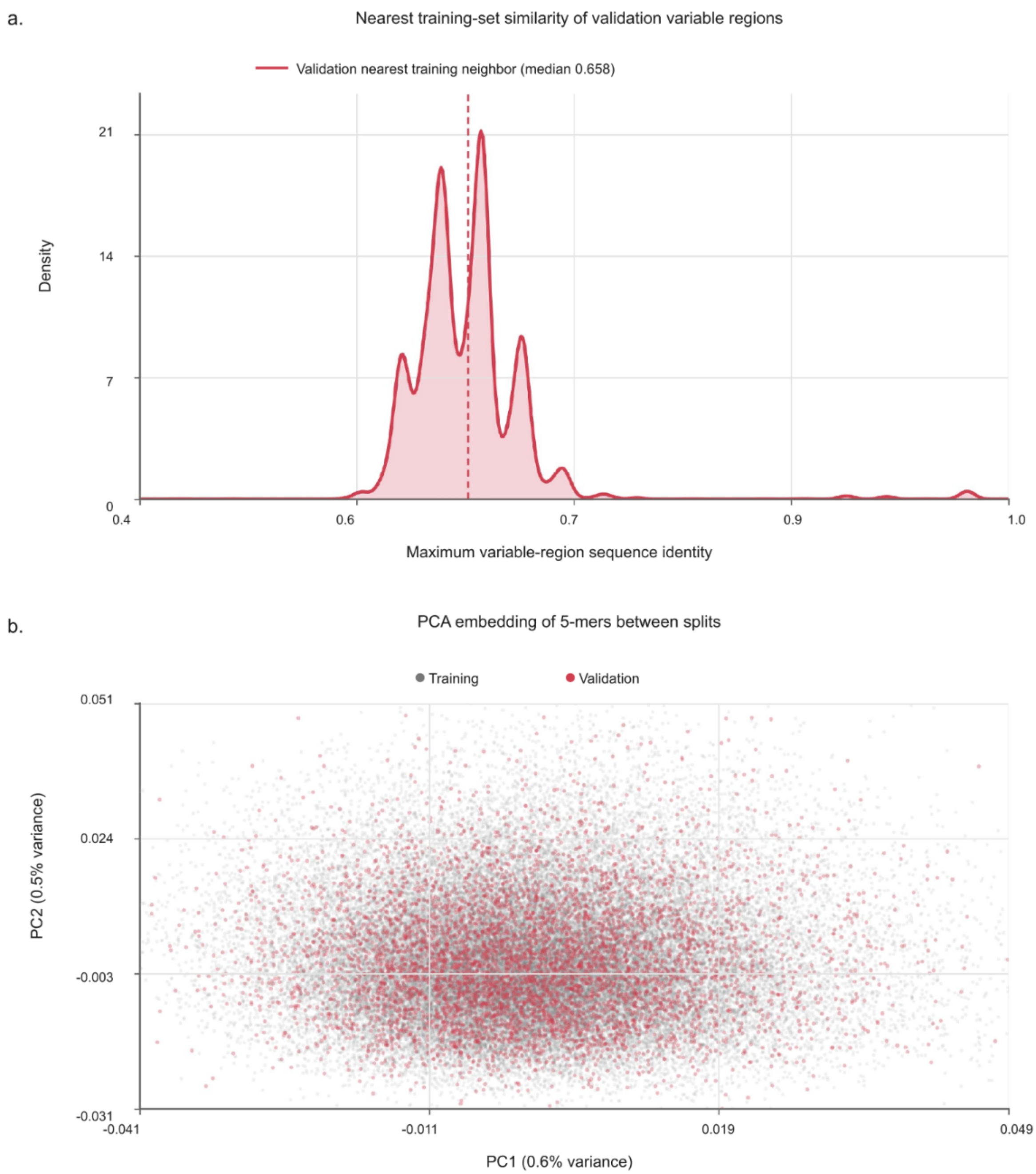

Supplementary Figure 1. Train–validation design-space similarity.

a. Density plot of nearest-neighbor sequence similarity between validation and training

variable regions. Three invariant catalytic-core segments (GCTGTCACCGG, TCCGGTCTGATGAGTCC, GGACGAAACAGC) were removed from each sequence to isolate Loop I and Loop II. Each of the 5,756 validation examples was compared exhaustively with all 51,804 training examples. For each pair, Levenshtein distances were calculated separately for the two variable loops, summed, and normalized by their combined aligned lengths; the maximum identity for each validation example was retained. The dashed line marks the distribution median (0.658). The training and validation sets contain 0 exact variable-region overlaps.

b. Principal component analysis of normalized 5-mer frequency embeddings calculated from Loop I and Loop II only, excluding 5-mers spanning the loop boundary. PCA was fitted on the 51,804 training examples, and the 5,756 validation examples were projected into the fitted space and overlaid. Training examples are shown in gray and validation examples in red. PC1 and PC2 explain 0.6% and 0.5% of training-set variance, respectively.

Supplementary Fig. 2

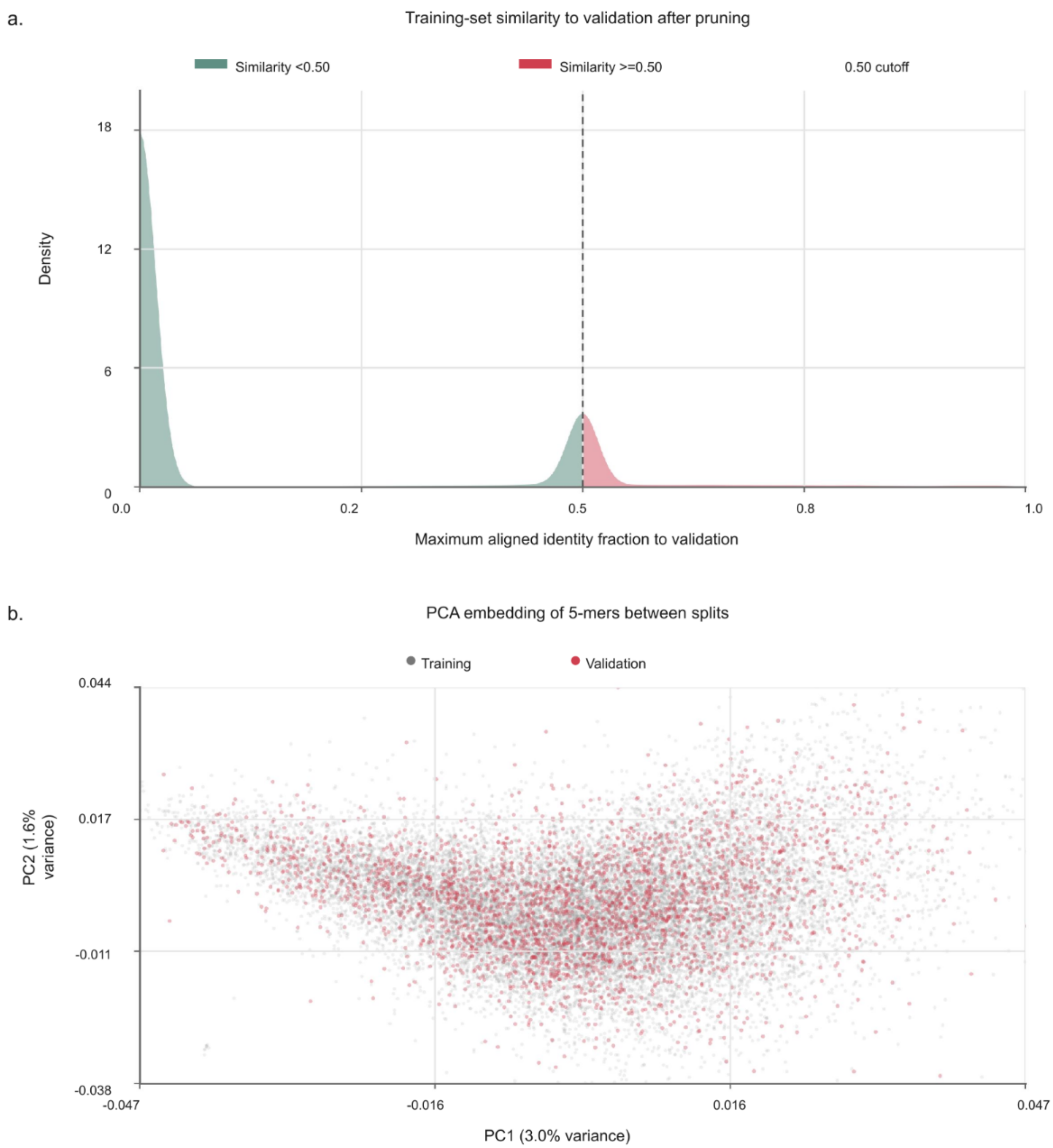

Supplementary Figure 2. Train–validation sequence-space comparison after similarity filtering.

a. Density of maximum local-alignment similarity from each original training viral insert to the

validation set. Constant cloning flanks and the 7-nt barcode were removed before comparison, leaving the 130-nt viral insert. Similarity is shown as aligned identical nucleotides divided by the full insert length. Training sequences with an MMseqs2 forward-strand Smith–Waterman local nucleotide alignment satisfying at least 50% nucleotide identity and at least 50% query and target coverage to any validation insert were removed before model training (4,669 of 23,484 original training rows). A single smoothed density is colored on each side of the 0.50 threshold; the dashed line marks the cutoff.

b. PCA embedding of normalized 5-mer frequency profiles calculated from the same 130-nt viral insert. PCA was fitted on the 18,815 retained training sequences, and the 2,935 validation sequences were projected into the fitted space and overlaid.

Supplementary Figure 3

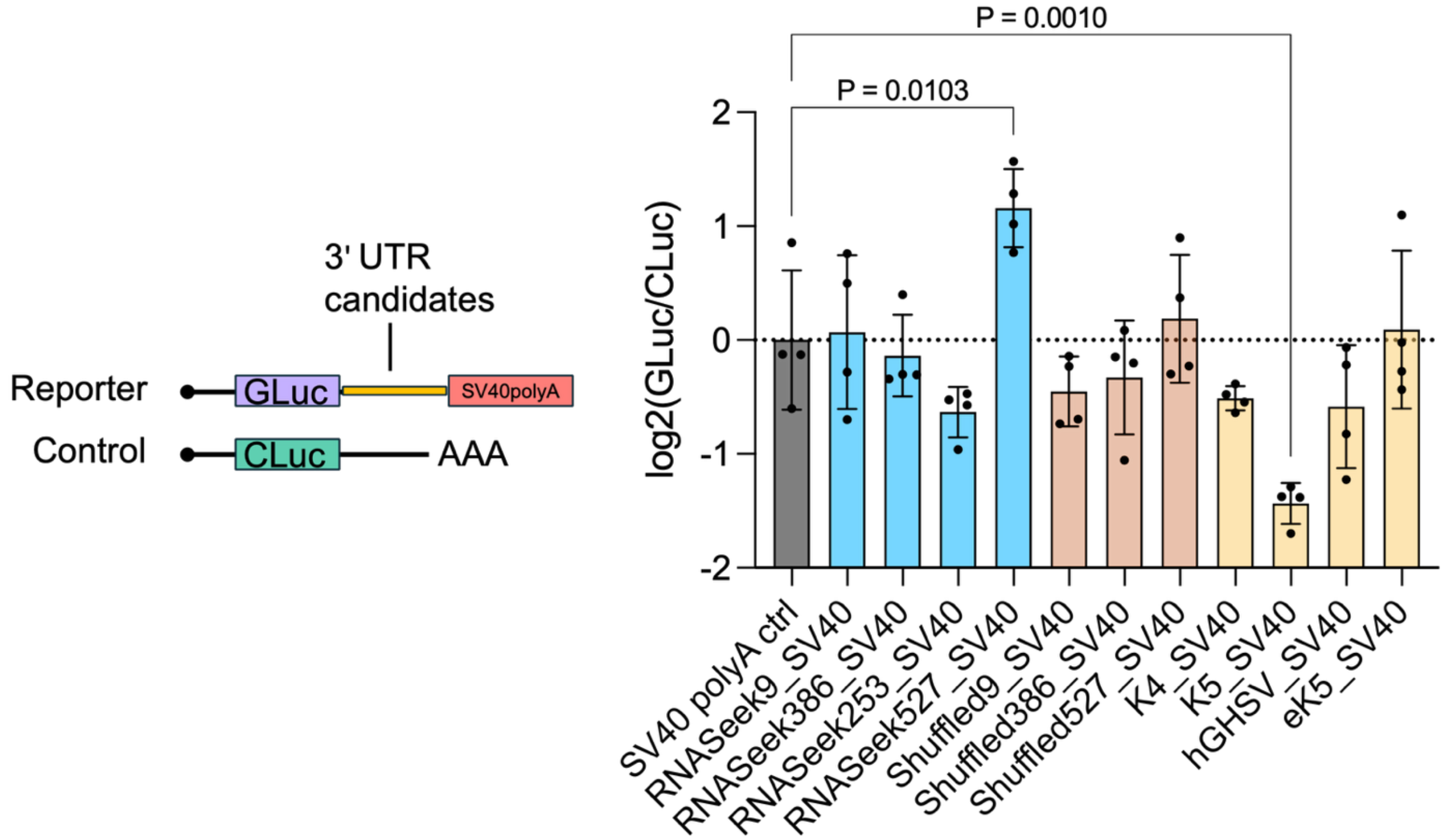

Supplementary Figure 3. Regulatory activity in combination with the SV40 poly(A) sequence. Reporter activity of RNASeek-generated 3′ UTR candidates (blue), their corresponding shuffled sequences (copper), and benchmark sequences from the training data (yellow) was measured in the Gluc/Cluc reporter system in which the sequences were inserted between the stop codon and the SV40 poly(A) sequence. Bars show mean reporter activity, with individual data points representing biological replicates. Statistical significance was assessed using two-sided unpaired *t*-tests with Dunnett's multiple-comparisons correction.
